# Clinical characteristics and risk factors for interventional management of abdominal trauma in pediatric patients admitted to a Pediatric Intensive Care Unit in Brazil

**DOI:** 10.1590/0100-6991e-2026001725-en

**Published:** 2026-03-19

**Authors:** LEILA COSTA VOLPON, CAMILA MAEKAWA DE ARAUJO, ANDERSON DE PAULA SOUZA, DAVI CASALE ARAGON, SANDRO SCARPELINI, ANA PAULA DE CARVALHO PANZERI CARLOTTI

**Affiliations:** 1 - Faculdade de Medicina de Ribeirão Preto da Universidade de São Paulo (Puericultura e Pediatria) - Ribeirão Preto - São Paulo - SP - Brasil; 2 - Faculdade de Medicina de Ribeirão Preto da Universidade de São Paulo (Cirurgia e Anatomia) - Ribeirão Preto - São Paulo - SP - Brasil

**Keywords:** Pediatrics, Laparotomy, Pediatric Emergency Medicine, Blood Transfusion, Multiple Trauma, Pediatria, Laparotomia, Medicina de Emergência Pediátrica, Transfusão de Sangue, Traumatismo Múltiplo

## Abstract

**Introduction:**

To investigate the clinical characteristics and risk factors associated with interventional treatment in critically ill pediatric patients with Intra-abdominal Trauma (IAT).

**Methods:**

This was a retrospective cohort study of patients younger than 18 years with IAT admitted to a Pediatric Intensive Care Unit (PICU) at a Brazilian emergency hospital between January 2015 and January 2024. Patients with hollow viscus injury were excluded. Demographic, clinical, treatment, and outcome data were collected from medical records. Patients were divided into two groups according to management strategy: interventional or conservative.

**Results:**

During the study period, 41 patients with IAT were admitted to the PICU; 35 were included in the study. Most patients were male (77.1%), with a median age of 9.6 years (range, 0.7-17 years), and more than half had Injury Severity Score (ISS) values greater than 25. Motor vehicle collisions were the most common mechanism of injury (68.6%). The most frequently injured organs were the liver (57.1%) and spleen (40%). Fifteen patients (42.9%) underwent interventional management: 11 (73.3%) surgical intervention and 4 (26.6%) interventional radiology. Hypotension on admission and the need for massive transfusion were identified as risk factors for interventional treatment. Two patients (5.7%) died; both underwent damage control laparotomy.

**Conclusions:**

In this cohort of critically ill pediatric patients with solid organ IAT, 42.8% required interventional management. Hypotension on admission and the need for massive transfusion were risk factors for interventional management in this population.

## RESUMO

**Introdução:**

Estudar as características clínicas e os fatores de risco de tratamento intervencionista em pacientes pediátricos gravemente doentes com trauma intra-abdominal (TIA).

**Métodos:**

Trata-se de estudo retrospectivo de coorte envolvendo pacientes com TIA com menos de 18 anos de idade admitidos em unidade de terapia intensiva pediátrica (UTIP) de hospital de emergência brasileiro, de janeiro/2015 a janeiro/2024. Foram excluídos pacientes com lesão de víscera oca. Dados demográficos, clínicos, de tratamento e de desfecho foram coletados dos prontuários médicos. Os pacientes foram divididos em dois grupos de acordo com o manejo: intervencionista ou conservador.

**Resultados:**

No período do estudo, 41 pacientes com TIA foram admitidos na UTIP; 35 foram incluídos no estudo. A maioria era do sexo masculino (77,1%), com idade mediana de 9,6 anos (variação de 0,7 a 17 anos), e mais da metade apresentavam valores de ISS superiores a 25. Acidente automobilístico foi o mecanismo de trauma mais comum (68,6%). Os órgãos mais frequentemente lesionados foram fígado (57,1%) e baço (40%). Quinze pacientes (42,9%) foram submetidos ao tratamento intervencionista: 11 (73,3%) à cirurgia e 4 (26,6%) à radiologia intervencionista. Hipotensão arterial na admissão e necessidade de transfusão maciça foram fatores de risco de tratamento intervencionista. Dois pacientes (5,7%) morreram; ambos foram submetidos à laparotomia de controle de danos.

**Conclusões:**

Nesta coorte de pacientes pediátricos gravemente doentes com TIA de vísceras sólidas, 42,8% necessitaram de tratamento intervencionista. Hipotensão arterial na admissão e necessidade de transfusão maciça foram fatores de risco de manejo intervencionista nesta população.

## INTRODUCTION

Intra-abdominal Trauma (IAT) occurs in 6% to 8% of pediatric trauma victims and is most commonly caused by motor vehicle collisions, bicycle accidents, and falls^1^. According to the most recent guidelines from the American Pediatric Surgical Association (APSA), more than 90% of intra-abdominal solid organ injuries in pediatric patients are managed nonoperatively, with interventional treatment indicated in the presence of hemodynamic instability and/or the need for massive transfusion^2^ ^,^ ^3^.

Previous studies have shown that physical examination findings, including the seatbelt sign, tachycardia, abdominal wall contusion associated with lumbar fracture, and free intra-abdominal fluid, are associated with the need for laparotomy following motor vehicle collisions^4^ ^-^ ^6^. However, there is a paucity of studies addressing the characteristics and management of critically ill patients with IAT requiring admission to a Pediatric Intensive Care Unit (PICU), particularly in the brazilian context.

Our objective was to investigate the clinical characteristics and risk factors for interventional management of pediatric patients with IAT admitted to the PICU of an emergency hospital in Brazil over 9 years.

## METHODS

This was a retrospective cohort study conducted in a medical-surgical PICU of a tertiary university hospital in Brazil. The study was approved by the Institutional Research Ethics Committee (#83069318.0.0000.5440). Informed consent was waived due to the retrospective nature of the study. All patients younger than 18 years of age with IAT admitted to the PICU between January 2015 and January 2024 were eligible for inclusion. Demographic and clinical data were collected from patient medical records, including injury characteristics, vital signs, laboratory and imaging findings, treatment, complications, and outcomes such as mortality and length of stay in the PICU and hospital. Patients with IAT who were not admitted to the PICU or who had a pre-established indication for operative management, such as hollow viscus injury, were excluded.

Patients were divided into two groups based on IAT-related management: conservative or interventional. The interventional group included damage-control laparotomy, definitive laparotomy, pigtail catheter insertion, and transcatheter arterial embolization performed by interventional radiology.

Injuries were classified according to the American Association for the Surgery of Trauma (AAST) organ injury grading scales. Fluid resuscitation was defined as the administration of >10mL/kg of crystalloid solution as bolus therapy in the prehospital setting or upon hospital admission for hemodynamic instability, characterized by clinical and laboratory signs of tissue hypoperfusion. Massive transfusion was defined as the administration of more than 40 mL/kg of blood products within the first 24 hours after trauma. Age-adjusted reference values for vital signs were based on Pediatric Advanced Life Support (PALS) recommendations^7^. Trauma severity was assessed using the Injury Severity Score (ISS) and the Clinical Abdominal Scoring System (CASS)^8^ ^,^ ^9^. ISS values greater than 15 were considered indicative of severe trauma^10^. CASS scores range from 5 to 15. A score ≥12 or ≤8 was associated with an overall accuracy of 94% for management decision-making in a previous study^9^.

### Statistical Analysis

Statistical analysis was performed using SAS version 9.2 (SAS/STAT User’s Guide 2008, version 9.2; SAS Institute, Cary, NC). Data are presented as median (interval) or number (%). Continuous variables were compared using the nonparametric Wilcoxon test, and categorical variables were compared using Fisher’s exact test or the chi-square test. To identify risk factors for interventional management, Relative Risks (RRs) and 95% Confidence Intervals (95% CIs) were obtained using log-binomial regression models. Initially, univariate log-binomial regression models were constructed to estimate crude RRs. Subsequently, multivariable log-binomial regression models were fitted to obtain adjusted RRs, with ISS included as a covariate. A significance level of 5% was adopted for all analyses.

## RESULTS

**Figure 1:**
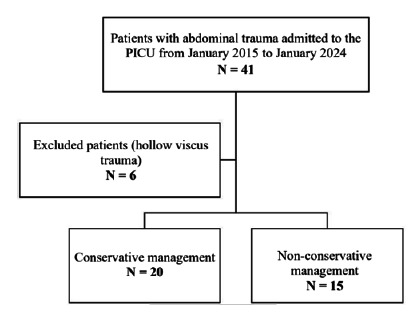
Study flowchart.

During the study period, 41 patients with IAT were admitted to the PICU; six were excluded due to hollow viscus injury. Thirty-five patients were included in the study: 15 (42.9%) underwent interventional management, and 20 (57.1%) received conservative management (Figure 1).

Demographic, clinical, and outcome data for the study population are presented in Table 1. In both groups, male patients predominated, and the most common mechanisms of injury were motor vehicle collisions and falls. The liver was the most commonly injured organ, followed by the spleen. Approximately one-third of patients sustained injuries to more than one intra-abdominal organ. All patients who underwent interventional management had ISS values greater than 15, compared with 70% of patients managed conservatively. Additionally, a significantly higher proportion of patients in the interventional group received a massive transfusion protocol.

**Table 1:** Demographic, clinical, and outcome data of the studied population.

Variables	All patients (n = 35)	Interventional Management (n = 15)	Conservative Management (n = 20)	p value
Age (Years)	9.6 (0.7-17)	12.1 (3-17)	8.5 (0.7-17)	0.41
Sex: male	27 (77.1)	11 (73.3)	16 (80)	0.7
ISS	26 (5-75)	33 (16-75)	25 (5-59)	0.28
ISS >15	29 (82.9)	15 (100)	14 (70)	0.03
CASS	9 (6-13)	9 (6-10)	9 (6-13)	0.74
Cause Automobile accident Fall Bicycle accident Direct impact	24 (68.6) 7 (20) 2 (5.7) 2 (5.7)	9 (60) 4 (26.7) 2 (13.3) 0 (0)	15 (75) 3 (15) 0 (0) 2 (10)	0.17
Injured organ Liver Spleen Kidney Pancreas	20 (57.1) 14 (40) 10 (28.6) 3 (8.6)	7 (46.7) 7 (46.7) 6 (40) 3 (20)	13 (65) 7 (35) 4 (20) 0 (0)	0.32 0.51 0.27 0.07
> 1 injured abdominal organ	12 (34.3)	7 (46.7)	5 (25)	0.28
Grade IV or V injury	15 (42.9)	8 (53.3)	7 (35)	0.17
TBI	16 (45.7)	5 (33.3)	11 (55)	0.31
Massive transfusion	8 (22.9)	7 (46.7)	1 (5)	0.01
Hypotension	6 (17.1)	5 (33.3)	1 (5)	0.06
Lactate (mmol/L)	2.8 (0.8-8.2)	3.5 (0.8-8.2)	2.2 (0.9-6.9)	0.3
INR	1.26 (0.91-2.1)	1.32 (1.07-2.1)	1.23 (0.91-1.73)	0.15
Fluid resuscitation	19 (54.3)	11 (73.3)	8 (40)	0.09
Length of stay in the PICU (days)	3 (1-30)	3 (1-24)	3 (1-30)	0.84
Length of hospital stay (days)	7 (2-72)	9 (3-72)	6.5 (2-50)	0.63
DEATHS	2 (5.7)	2 (13.3)	0 (0)	0.17

Data were expressed as median (range) or n (%). ISS, Injury Severity Score; CASS, Clinical Abdominal Scoring System; TBI, Traumatic Brain Injury; INR, International Normalized Ratio; PICU, Pediatric Intensive Care Unit.

Among the 15 patients who underwent interventional management, 11 (73.3%) underwent surgical intervention, and 4 (26.6%) underwent interventional radiology procedures (transarterial embolization). The surgical procedures performed included definitive laparotomy (n = 7), damage control laparotomy (n = 3), and pigtail catheter insertion (n = 1).

Risk factors for interventional treatment were hypotension on admission and the need for massive transfusion, with adjusted RRs of 2.5 and 3.15, respectively, after adjustment for ISS (Table 2).

**Table 2:** Risk Factors for Interventional Management.

Variables	Conservative Management	Interventional Management	Relative Risk (95% CI)	Relative Risk* Adjusted
Hypotension on admission				
No	19 (65.5)	10 (34.5)	reference	reference
Yes	1 (16.7)	5 (83.3)	2.41 (1.30; 4.48)	2.50 (1.04; 6.01)
Need for massive transfusion				
No	12 (75)	4 (25)	reference	reference
Yes	8 (42.1)	11 (57.9)	2.31 (0.91; 5.87)	2.36 (0.86; 6.49)
Need for massive transfusion				
No	19 (70.4)	8 (29.6)	reference	reference
Yes	1 (12.5)	7 (87.5)	2.95 (1.57; 5.59)	3.15 (1.49; 6.67)
CASS				
≤8	8 (53.3)	7 (46.7)	reference	reference
>8	12 (60)	8 (40)	0.85 (0.40; 1.84)	-
ISS				
≤25	11 (64.7)	6 (35.3)	reference	reference
>25	9 (50)	9 (50)	1.41 (0.64; 3.13)	-

CASS, Clinical Abdominal Scoring System; ISS, Injury Severity Score. *For the calculation of adjusted relative risks, the ISS was considered as a covariate in a multiple log-binomial model.

Postoperative complications were observed in only one patient, a victim of a motor vehicle collision with hepatic and renal injuries, who underwent damage control laparotomy and subsequently developed a subdiaphragmatic abscess. No other patients in the interventional management group experienced complications.

Overall mortality in our cohort was 5.7%. Two patients with very high ISS values (75 and 48) who underwent damage control laparotomy died during their PICU stay. The causes of death were severe traumatic brain injury in one patient and septic shock in the other, who also sustained spinal cord and orthopedic injuries.

## DISCUSSION

Our study included critically ill pediatric patients with IAT and solid organ injury resulting from severe multisystem trauma, with a median ISS of 26. The most common mechanism of injury was motor vehicle collision, which contrasts with studies involving emergency department populations, including patients with mild injuries, that report falls as the leading mechanism of IAT^11^ ^,^ ^12^.

More than 40% of patients in our study required interventional treatment. Although nonoperative management of solid organ injuries has become the standard of care, patients with severe trauma may require a more aggressive approach. The literature on pediatric trauma patients is limited compared with adult populations, particularly with respect to surgical cases in developing countries^13^ ^,^ ^14^. Our data demonstrate that more severely injured patients have higher rates of failure of conservative management compared with other studies that included patients across a broader spectrum of injury severity^15^ ^-^ ^18^. A multicenter study of patients with IAT and a median ISS of 16, treated at 14 Level I pediatric trauma centers in the United States of America, found that fewer than one-fifth required acute intervention (angiographic embolization or laparotomy)^19^. Brazilian data on patients with IAT admitted to the PICU reported operative management in 28% of cases, although ISS values for that population were not specified^14^. We emphasize that our population represents a cohort of severely injured patients requiring admission to the PICU of a tertiary trauma referral center. Previous studies have demonstrated that severe solid organ injuries and traumatic brain injury, in combination with multisystem trauma, are associated with the need for ICU admission in pediatric patients with blunt abdominal trauma^20^. Furthermore, evidence suggests that ICU admission is more frequent among patients with abdominal trauma and multiple organ injury who require interventional management compared with those treated conservatively^21^. Thus, the higher rate of interventional management observed in our study may be explained by the inclusion of pediatric patients with IAT associated with severe trauma requiring PICU admission.

We identified hypotension on admission and the need for massive transfusion as risk factors for interventional management of IAT in critically ill pediatric patients with parenchymal organ injury. In fact, pediatric abdominal trauma management guidelines identify hemodynamic status as the primary determinant in decision-making for interventional management, as well as the extent of blood loss^3^. However, the hemodynamic instability frequently observed in polytrauma patients may result not only from bleeding due to intra-abdominal organ injury but also from the systemic inflammatory response triggered by trauma, further complicating decisions regarding interventional management of IAT.

In our study, elevated CASS values were not identified as a risk factor for interventional treatment. This score was proposed as a screening tool to identify the need for laparotomy in patients with IAT. However, a previous study demonstrated that CASS had low specificity and, consequently, limited ability to predict the need for surgical intervention in patients with blunt IAT^22^.

Another study showed that grade 4 or 5 abdominal organ injuries, regardless of the organ involved, combined injuries, ISS >25, bicycle-related trauma, and pancreatic injury were associated with a higher risk of failure of conservative management of IAT^23^. Consistent with these findings, we observed that all patients injured in bicycle accidents and all patients with pancreatic injury in our cohort required interventional treatment.

In our study, four patients with IAT were successfully treated with arterial embolization without complications. Only one of these patients presented with signs of hemodynamic instability at initial evaluation, but all of them had imaging evidence of arterial bleeding on computed tomography. Recent guidelines consider arterial embolization a useful tool in the management of solid organ injuries in patients with arterial contrast blush on imaging and hemodynamic compromise due to ongoing bleeding^3^ ^,^ ^24^.

Damage control laparotomy was performed in 3 (8.5%) patients in our study. Despite its widespread acceptance in adult trauma care, few reports have described its use in severely injured pediatric patients^25^. In South Africa, 11% of emergency laparotomies for pediatric trauma were damage control laparotomies^26^. Villalobos et al. described a cohort of 56 damage control laparotomies in patients with high ISS, reporting a mortality rate of 45%^27^. Both patients who died in our cohort underwent damage control laparotomy. There is evidence that damage control laparotomy is a risk factor for mortality in surgically managed blunt abdominal trauma^28^.

The strength of this study lies in the inclusion of pediatric patients with IAT and severe multisystem trauma requiring PICU admission, as well as the cohort analysis of this profile in Brazil, where published data are scarce. Study limitations include the relatively small sample size, its retrospective design, and the single-center setting, which may limit the generalizability of the findings.

## CONCLUSIONS

Approximately 43% of pediatric patients admitted to the PICU with IAT required interventional treatment. Hypotension and the need for massive transfusion on admission were risk factors for interventional management in this population.
